# Caregiver Perception of Weight Status in 5-Year-Old Children From a Community of High Socioeconomic Deprivation in New Zealand

**DOI:** 10.3389/fpubh.2022.641418

**Published:** 2022-06-30

**Authors:** Éadaoin M. Butler, José G. B. Derraik, Alison Burge, Wayne S. Cutfield, Alison Leversha

**Affiliations:** ^1^Liggins Institute, University of Auckland, Auckland, New Zealand; ^2^A Better Start – National Science Challenge, University of Auckland, Auckland, New Zealand; ^3^Department of Paediatrics: Child and Youth Health, Faculty of Medical and Health Sciences, University of Auckland, Auckland, New Zealand; ^4^Department of Women's and Children's Health, Uppsala University, Uppsala, Sweden; ^5^Environmental - Occupational Health Sciences and Non-Communicable Diseases Research Group, Research Institute for Health Sciences, Chiang Mai University, Chiang Mai, Thailand; ^6^Starship Community Services, Auckland District Health Board, Auckland, New Zealand

**Keywords:** BMI, health, Māori, obesity, overweight, Pacific, recognition

## Abstract

**Background:**

Early childhood obesity is highly prevalent in Aotearoa New Zealand (NZ). Little is known about caregiver perception of children's weight status among those living in areas of high socioeconomic deprivation, particularly Māori and Pacific children.

**Aims:**

To explore caregiver perception of weight status among children starting school in areas of high socioeconomic deprivation and examine potential associations between the child's body mass index (BMI) z-score and their caregiver's perception of their child's body size or health.

**Methods:**

Participants were 5-year-old children living in a community of high socioeconomic deprivation and their caregivers. Children had their weight and height measured. BMI *z*-scores were calculated according to World Health Organization standards. Caregivers were asked to assess their child's BMI and health status, and choose a silhouette that best represented their child's body size.

**Results:**

One hundred and six children (>75% Māori or Pacific) were included. Over half (58%) had overweight or obesity, with only 16% correctly perceived by their caregiver as overweight. These children tended to have higher BMI *z*-scores than those not correctly perceived as overweight. Caregivers chose larger silhouettes to represent children's body sizes as children's BMI *z*-scores increased. There was no discernible association between children's BMI *z*-scores and caregiver perception of children's health.

**Conclusions:**

Caregivers appeared to judge their child's body size in comparison to other children. The normalization of childhood obesity and infrequent caregiver recognition of this condition in children in communities with a high prevalence may impact the uptake and efficacy of intervention initiatives.

## Introduction

Overweight in early childhood is estimated to affect 38 million children worldwide (1). In Aotearoa New Zealand (NZ), despite some recent evidence for a slight decline, the prevalence of overweight/obesity in early childhood remains high (>30%) (2). Childhood obesity tracks into later life (3, 4), and is associated with increased risk of comorbidities both in childhood and adolescence (5), and adulthood (6). Thus, the early prevention and treatment of childhood obesity remain important public health concerns in NZ and elsewhere.

Much interest has been placed on parental perception of their child's weight status, based on the belief that accurate parental perception of childhood overweight or obesity will facilitate better weight outcomes for children (7–12). However, internationally, the majority of studies have reported parents do not accurately identify overweight or obesity in children, and frequently under-estimate their weight status (7–11). Common factors influencing parental perception of children's weight status include: child's age and sex, parental education, and socioeconomic status (7–11). Parental perception of their child's overall health and lifestyle may be important too, with qualitative research suggesting parents of young children do not feel concerned by their child's weight status if they perceive them to be in good health and/or engaged in regular physical activity (13–16). Additionally, systematic reviews have reported improved parental accuracy in studies which use visual assessments such as silhouettes, rather than verbal assessments requiring parents to select the words “overweight” or “obese”, which are potentially more subject to stigma (9, 10).

Three previous studies have examined parental perceptions of their children's weight status in NZ (17–19), with all reporting high rates of under-perception of excess weight status among parents of children with overweight or obesity (17–19). Of these studies, two did not break down parental perception by ethnicity (18, 19), while the other reported that misperception did not differ by ethnicity (17). However, children included in the latter study were predominantly of NZ Europeans, while only 1 in 5 lived in areas of high socioeconomic deprivation (17). A greater proportion of children of Māori (indigenous people of NZ) and Pacific ethnicity have early childhood obesity (20.0 and 30.2%, respectively) compared to European or Asian children (12.7 and 8.1%, respectively) (2). Furthermore, obesity affects <10% of 5-year-olds living in the least deprived areas of NZ, while it affects just over 20% of those living in the most deprived areas (2).

Therefore, the aim of this study was to explore the accuracy of caregiver perception of weight status at school entry among children living in an Auckland community of high socioeconomic deprivation (predominantly Māori and Pacific). We also aimed to examine potential associations between children's body mass index (BMI) *z*-scores and caregiver perception of children's body size when assessed with silhouettes or verbally, and their perception of their child's health.

## Methods

Participants were drawn from the Welcome-to-School Study, which has been described previously (20). Briefly, the study recruited 5-year-old children (and their caregivers) at school entry living in a central Auckland community of high socioeconomic deprivation (20). Children underwent comprehensive health, developmental, educational, and social assessments with appropriate referrals and linkages made if any issues were identified (20). All children had their heights and weights measured while barefoot and wearing light clothing by a research nurse, following a standardized protocol. Height was measured using a SECA 217 portable stadiometer (SECA, Hamburg, Germany) to the nearest mm, while weight was measured with a SECA 874 digital scale to the nearest 0.01 kg. Assessments occurred at either the child's home or school, according to caregiver preference.

Caregivers were interviewed face-to-face about their child's health and development by the research nurse using a standardized questionnaire (20). The specific questions included in this study were those asking caregivers about their perceptions of their child's weight (underweight, normal weight, overweight, or don't know), body size (visual assessment), and current health (poor, fair, good, very good, excellent, or don't know) (Supplementary Material). The tool used for visual assessment of child's body size was sourced from a previous study about overweight in Latino preschoolers (21), and was composed of 12 line drawn silhouettes of girls and boys (six each) ranked from “C” (representing relatively thin) to “H” (representing severe obesity) (Supplementary Material). Caregivers were not informed of weight status attributed to the silhouettes.

Demographic information was collected, including caregiver education level, and child's age, sex, and ethnicity. Caregiver education was stratified as high school or less, certificate/diploma, or University degree. Children's ethnicity was classified using the Statistics NZ order of prioritization (22), with the exception that all children not classified as either Māori or Pacific were classified as “Other” due to their small number. The participant's home address was used to derive their level of socioeconomic deprivation (NZDep2013 score), ranging from 1 (least deprived) to 10 (most deprived) (23), which were stratified into quintiles.

One hundred and twenty-one children were enrolled and examined for the Welcome-to-School Study over the 12-month study period, which equated to approximately: 75% of the eligible children starting school in the area. Only those with available data on weight, height, and caregiver perception of weight status were included in the present study (*n* = 106, 88% of study participants or 67% of the total cohort of children starting school).

### Statistical Analyses

BMI was calculated and transformed into age- and sex-adjusted *z*-scores as per World Health Organization (WHO) standards (24). The child's BMI status was subsequently defined as: normal weight or underweight [BMI *z*-score < 1.036 (i.e., <85th percentile)], overweight [≥1.036 but <1.645 (i.e., <95th percentile)], or obesity (*z*-score ≥ 1.645) (25).

The BMI of children with overweight or obesity correctly perceived as such by their caregiver were compared to those who were not with a two-sample *t*-test. BMI *z*-scores among children identified as Māori, Pacific, or “Other” were compared using a general linear model.

The associations between the child's BMI *z*-score and the caregiver's choice in the picture scale or their perception of the child's health were examined using non-parametric Spearman's rank correlations, with results reported as the Spearman's rho coefficient (ρ) and the respective 95% confidence interval (CI) and *p*-value. Differences in the caregiver ranking of children along the picture scale according to ethnicity and gender were compared using non-parametric Kruskal-Wallis tests.

Group summary data are provided in the text as means ± standard deviations, except for picture scale data reported as medians, quartile 1 (i.e., 25th percentile), and quartile 3 (i.e., 75th percentile). Data were analyzed using SPSS v25 (IBM Corp, Armonk, NY, USA) and Minitab v21 (Pennsylvania State University, State College, Pennsylvania, USA). All statistical tests were two-sided with significance set at *p* < 0.05. Figures were created using GraphPad Prism v8.2 (GraphPad Software Inc., San Diego, CA, USA).

## Results

### Participant Characteristics

There were 117 children with BMI data in the Welcome-to-School Study. However, caregiver perception of their child's weight status was not available for nine participants, and two caregivers responded “don't know”; leaving 106 children in this study with valid data (Table 1).

**Table 1:** Sociodemographic characteristics of participants.

*n* ^a^			106
Child	Age (years)		5.3 ± 0.2
	Sex	Female	49 (46.2%)
	Ethnicity	Māori	20 (18.9%)
		Pacific	62 (58.5%)
		Other	24 (22.6%)
	BMI status	Underweight/normal weight	45 (42.5%)
		Overweight	22 (20.8%)
		Obesity	39 (36.8%)
	BMI *z-*score		1.48 ± 1.75
Caregiver	Education	High school or less	49 (48.5%)
		Certificate/diploma	36 (35.6%)
		University degree	16 (15.8%)
	Socioeconomic deprivation (quintile)^b^	1 (least deprived)	1 (1.1%)
		2	1 (1.1%)
		3	6 (6.4%)
		4	10 (10.6%)
		5 (most deprived)	76 (80.9%)

*BMI, body mass index*.

*Data are n (%) or mean ± SD*.

a *n = 106, except for “Education” (n = 101) and “Socioeconomic deprivation” (n = 94)*.

b *Socioeconomic deprivation was measured using the NZDep2013 (23)*.

*Child's weight status was defined based on body mass index (BMI) z-scores standardized for age and sex as per World Health Organization standards (24), as follows: overweight, BMI ≥ 85th to <95th percentile (z-score ≥ 1.036 and <1.645); and obesity, BMI ≥ 95th percentile (z-score ≥ 1.645) (25)*.

Just under half were female (46%). More than half (*n* = 61; 58%) had overweight or obesity (Table 1). Few caregivers (16%) had a University education (Table 1). Over 80% lived in areas with the highest quintile of socioeconomic deprivation (Table 1). Almost all questionnaires were completed by a female primary caregiver (mostly the child's mother).

### Caregiver Perception of Child's Weight Status

Table 2 summarizes caregiver perceived vs. actual weight status of children. There were no children of underweight, normal weight, or overweight status perceived as overweight by their caregiver (Table 2). Only 10 out of 39 (26%) children with obesity were perceived as having overweight, while three children with obesity were perceived as underweight (Table 2). Supplementary Table 1 shows that the proportions of girls (23.1%) and boys (26.9%) with obesity considered overweight by their caregiver were similar.

**Table 2:** Caregiver perceived vs. actual weight status of the child (*n* = 106).

	**Child's weight status**		
**Caregiver perception**	**Normal weight/underweight**	**Overweight**	**Obesity**
n	45	22	39
Underweight	7 (15.6%)	2 (9.1%)	3 (7.7%)
Normal weight	38 (84.4%)	20 (90.9%)	26 (66.7%)
Overweight	Nil	Nil	10 (25.6%)

*Data are n (%). Child's weight status was defined based on body mass index (BMI) z-scores standardized for age and sex as per World Health Organization standards (24), as follows: overweight, BMI ≥ 85th to <95th percentile (z-score ≥1.036 and <1.645); and obesity, BMI ≥ 95th percentile (z-score ≥ 1.645) (25)*.

**Figure 1:**
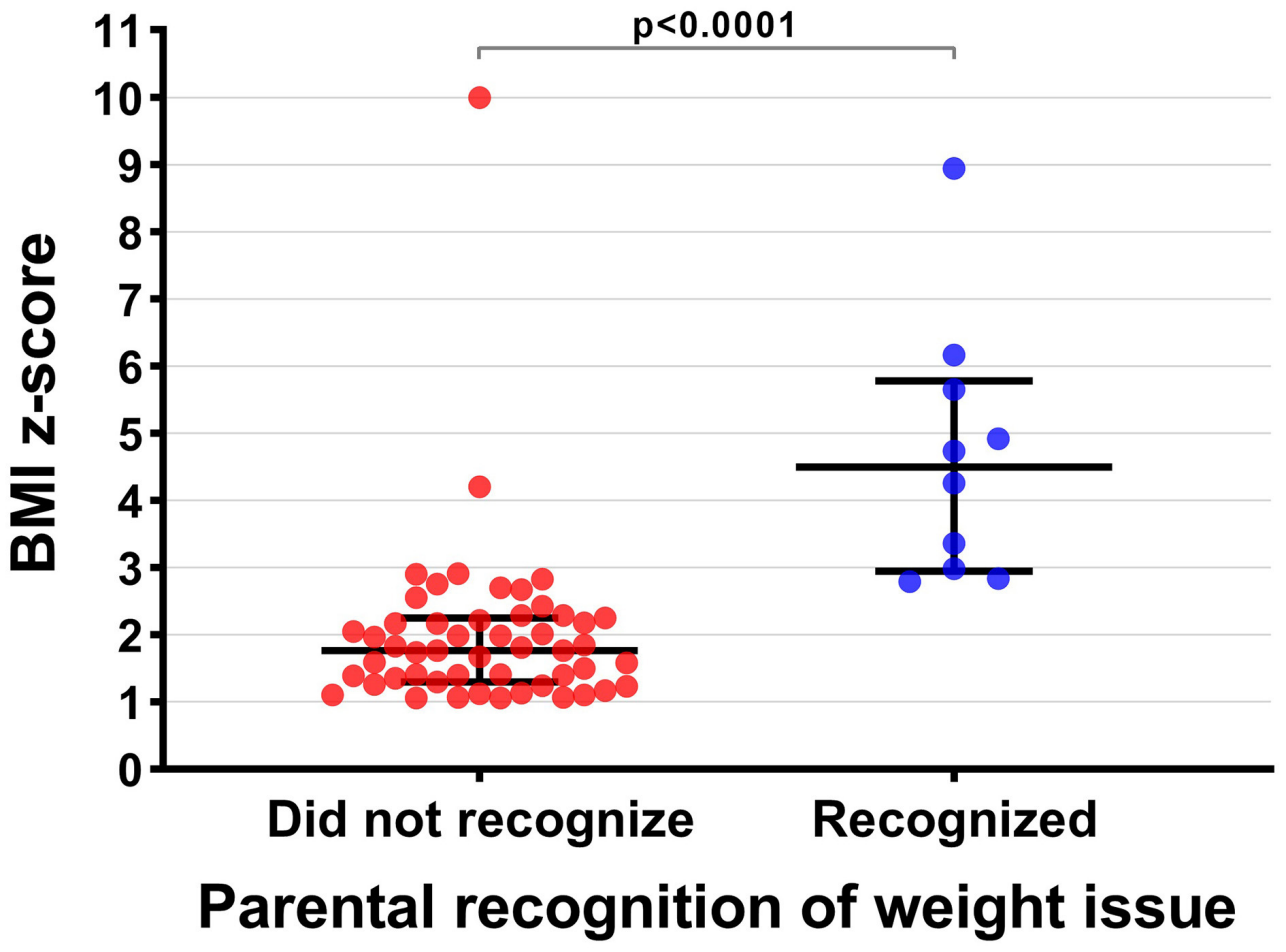
Caregiver recognition of overweight in children with overweight or obesity and the children's respective body mass index (BMI) z-scores (*n* = 61). BMI z-scores were standardized for age and sex as per World Health Organization standards (24). Horizontal black bars represent the median and the interquartile range, while the *p*-value for the comparison between groups was derived from a *t*-test.

Further highlighting the association between caregiver perception of child's weight status and BMI *z*-score, children with overweight or obesity correctly recognized as such by their caregiver tended to have higher BMI *z*-scores than those who were not (4.66 ± 1.92 vs. 2.00 ± 1.32, respectively; *p* < 0.0001) (Figure 1). One notable exception was a boy with a confirmed BMI *z*-score of 10.06 perceived as underweight by his mother.

**Figure 2:**
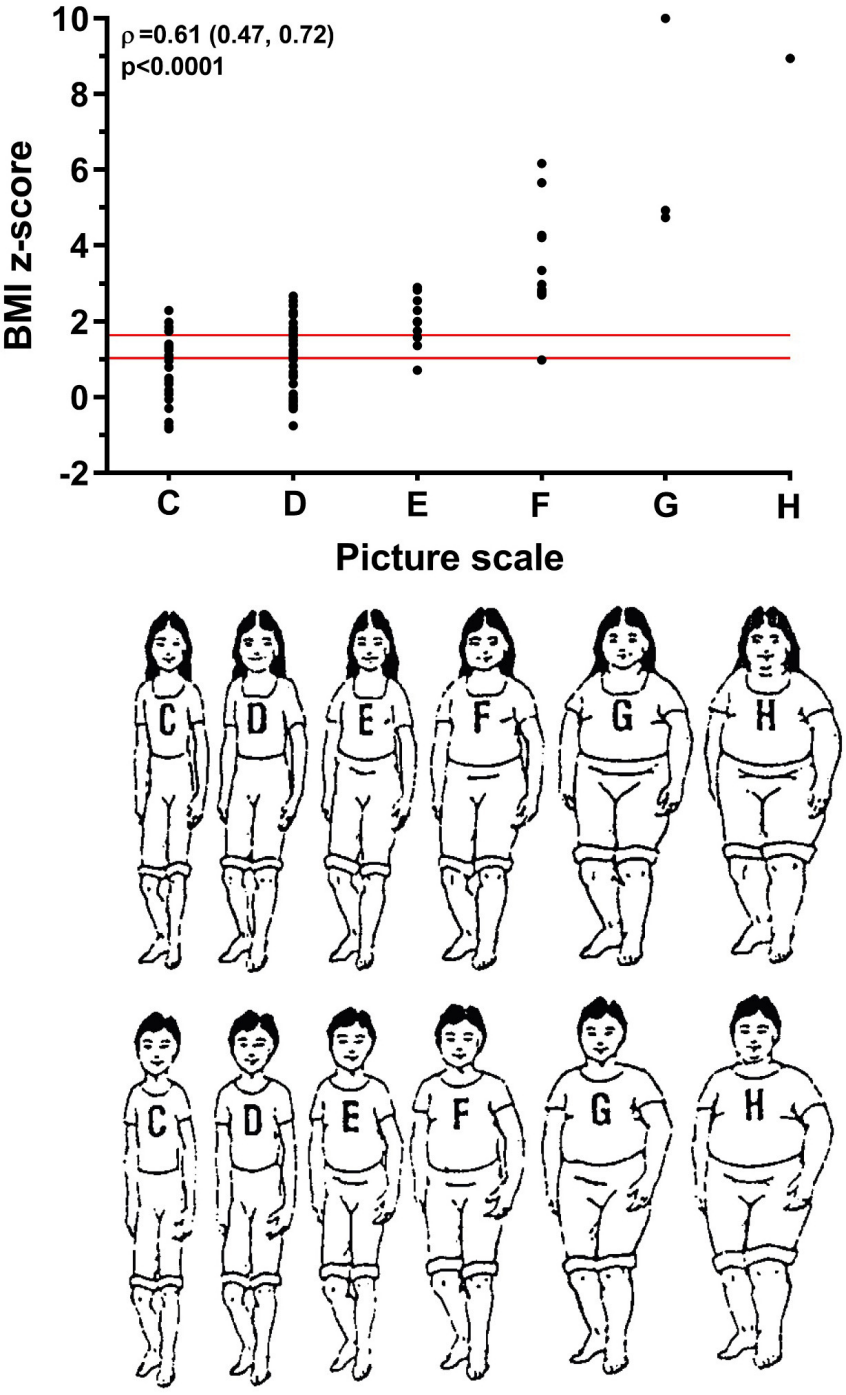
Caregiver choice of silhouette and child body mass index (BMI) *z-*score (*n* = 103). Children's BMI z-scores were standardized for age and sex as per World Health Organization standards (24). The two red lines parallel to the *x*-axis represent the thresholds for overweight and obesity, defined as: overweight, BMI ≥ 85th to <95th percentile (z-score ≥1.036 and <1.645); and obesity, BMI ≥ 95th percentile (z-score ≥ 1.645) (25). Figure of silhouettes reproduced with permission from Kersey et al. (21). The association between the two parameters is reported as the Spearman's rank correlation coefficient (ρ) and the respective 95% confidence interval and *p*-value.

Figure 2 shows caregiver choice of a silhouette in relation to children's BMI *z*-scores. Not surprisingly, caregivers tended to choose larger silhouettes as their children's BMI *z*-scores increased (ρ = 0.61; *p* < 0.0001) (Figure 2). However, many caregivers selected a slim silhouette for their children, despite them having overweight or obesity (Figure 2). These findings were largely unchanged regardless of whether children were identified as Māori, Pacific, or Other (Supplementary Figure 1).

The only observable ethnic differences were lower BMI *z*-scores among children identified as “Other” ethnicities (0.46 ± 0.88) compared to children identified as Māori (1.37 ± 1.45; *p* = 0.046) or Pacific (1.90 ± 1.85; *p* < 0.0001). Nonetheless, while caregivers of Pacific children were the only ones choosing silhouettes at the upper end of the visual scale (Supplementary Figure 1), there were no differences in the ranking of their children among the three ethnic groups (*p* = 0.97). When results were examined by sex, the only discernible differences were higher BMI *z*-scores in boys (1.91 ± 1.95) than in girls (1.00 ± 1.20; *p* = 0.003), with corresponding larger silhouettes chosen by caregivers of boys [2 (2, 3)] compared to girls [2 (1, 2); *p* = 0.046] (Supplementary Figure 2).

### Caregiver Perception of Child's Weight vs. Child's Health Status

There was no correlation between caregiver perception of their child's health status and the child's BMI *z*-score [ρ = 0.02 (95% CI −0.18, 0.21); *p* = 0.85], with many children across the BMI range considered to be healthy (Supplementary Figure 3). In fact, very few children were deemed to have “poor” or “fair” health, and several children with very severe obesity (BMI z-score 4.20–10.06) were considered to have “very good” or “excellent” health (Supplementary Figure 3). Of the 10 children with obesity who were correctly recognized as having “overweight”, only one was considered to have “fair” health, while the rest were considered to have “good” (*n* = 3), “very good” (*n* = 3), or “excellent” (*n* = 3) health.

## Discussion

In a population of 5-year-old children in NZ living in an Auckland suburb of high socioeconomic deprivation, over half had overweight or obesity [compared to one in three overall in the country (2)], but few were perceived as overweight by their caregivers. It was uncommon for caregivers of children with overweight or obesity to choose larger silhouettes as representative of their child's body size, even though children correctly perceived as overweight tended to have higher BMI *z*-scores than those not perceived as such. In addition, we observed that caregiver perception of children's health status was not associated with children's BMI *z*-scores.

Similar to previous research in NZ (17–19) and elsewhere (7–11), caregiver recognition of overweight in children with overweight or obesity was infrequent in our study, even though Children perceived as overweight tended to be on the higher end of the BMI *z*-score distribution. It may be that the high prevalence of obesity in children and adults in communities represented by our study population [i.e. large proportions of Māori or Pacific people and those living in areas of high socioeconomic disadvantage (2, 26)], has created a “normalizing” effect (12), whereby overweight or obesity is so common it is considered the norm, so that only children with exceptionally high BMI *z*-scores may be perceived to carry excessive weight. Indeed, previous longitudinal studies have reported increased frequency of parental underestimation of weight status in children with overweight co-occurring with an increased prevalence of childhood obesity in a given population (12). Our findings from the silhouette assessment provide further evidence to support the normalization theory. There was a clear pattern of caregivers choosing increasingly larger silhouettes to represent their child's body size as children's BMI z-scores increased. Of note, many caregivers of children with a BMI *z*-score on the lower end of the obesity range chose slim silhouettes for their children. Therefore, it seems likely that caregiver judgment was influenced by their perception of their child's body size compared to other children in their community, rather than their child's BMI status *per se*. This phenomenon has been reported in different populations (12) and was consistent across the ethnicities in our study.

There was no apparent link between caregiver perception of children's health status and their BMI *z*-scores, with children across the BMI z-score range considered to be in “very good” or “excellent” health. This disconnect between weight status and health has been reported previously, particularly among caregivers of young children who point to other indicators of their child's health, including their happiness (14, 27, 28), dietary intake (14, 15), or levels of physical activity (14–16, 27). In NZ, Māori caregivers of children aged 5 years and younger believed that once children started school, they would burn off any excess fat through increased physical activity (27). However, elsewhere, parents of 7-year-old children were more accurate in recognizing their child's overweight or obesity when they perceived their health as poor (29). Indeed, parental recognition of overweight or obesity is more accurate in older than younger children (7, 9–11). Therefore, a link between caregiver perception of health and weight may not be seen among caregivers of children soon after starting school, such as those in our study, as weight-related comorbidities are less of immediate concern for them.

### Implications for Public Health

Our study has shown that in this community with a high prevalence of overweight or obesity, only a few children with very high BMI *z*-scores were perceived as overweight. Therefore, if caregiver recognition of excessive weight in their child is deemed important for early intervention, this may be challenging in communities with a high prevalence of childhood obesity where normalization and infrequent caregiver recognition of the condition may impact uptake and efficacy of interventions.

Theoretically, it would make sense that caregiver recognition of a weight issue in children would be followed by intervention. To this end, public health interventions in the UK and US have focused on providing caregivers of school children with a BMI report card so that they are aware of their child's weight status (30). In NZ, caregivers of children identified as “extremely overweight” at the B4School Check (a comprehensive check of the development and growth of children aged 4–5 years) are informed of their child's weight status, and their child is referred for weight management (31). However, longitudinal studies not involving any form of intervention have reported increased weight gain among children who have been recognized as having overweight or obesity by their parents (32–34). Furthermore, BMI report cards have not been as effective as hoped; (30) in the UK, for example, there were minimal parental-reported changes in health behaviors among parents who were informed that their child was above a healthy weight (35). Therefore, as we previously concluded (36), it seems unlikely that caregiver recognition of childhood obesity alone is sufficient for behavioral change. Supportive holistic interventions are needed to assist families in achieving a healthy weight for their children. Future research should also consider the longitudinal impact of accurate caregiver perception of children's weight status on childhood weight gain in the NZ context.

### Limitations and Strengths

Our study is limited by the relatively small number of participants, restricting our ability to generalize our findings to a wider population. However, our focus on children living in a community of high socioeconomic deprivation, in particular of Māori or Pacific ethnicity, and our sample comprising more than two thirds of the cohort of children starting school in a community of interest are key strengths. This is important given the higher prevalence of early childhood obesity among children in these groups in NZ (2). In addition, the images used for caregiver visual assessment of child body size were not culturally adapted to caregivers of Māori or Pacific preschool children. Still, their use meant that our study did not solely rely on a scale using words, which are more likely to be subject to misreporting due to stigma (9, 10).

### Conclusions

In this sample of predominantly Māori or Pacific children living in an urban NZ community of high socioeconomic deprivation, few (16%) were correctly perceived as overweight by their caregivers. Caregivers appeared to judge their child's body size in comparison to other children in their community rather than by their actual weight status, potentially contributing to the infrequent recognition of overweight. There did not appear to be a link between children's BMI *z*-scores and caregiver perception of children's health for those included in our study. The normalization of childhood obesity in communities with a high prevalence may impact the uptake and efficacy of intervention initiatives.

## Data Availability Statement

The datasets presented in this article are not readily available because of the strict conditions of the ethics approval. However, the anonymised data on which this article was based could be made available to other investigators upon bona fide request, and following all the necessary approvals (including ethics approval) of the detailed study proposal and statistical analyses plan. Requests to access the datasets should be directed to AL, alisonl@adhb.govt.nz.

## Ethics Statement

Welcome-to-School Study was approved by the Central Disability Ethics Committee of the NZ Ministry of Health (15/CEN/224/AM04). Written informed consent to participate in this study was provided by the participants' legal guardian/next of kin.

## Author Contributions

Welcome-to-School Study: AL was the principal investigator and obtained funding. AL and AB contributed to study design. AB enrolled participants, performed assessments, and collected and curated data. Current study: WC and JD obtained funding. ÉB, JD, WC, and AL contributed to study design. JD and ÉB were responsible for data curation and analyses. ÉB wrote the manuscript with assistance from JD and critical input from WC, AL, and AB. All authors have approved the submission of the manuscript in its final form.

## Funding

ÉB was supported by a scholarship from A Better Start – National Science Challenge, which was funded by the NZ Ministry of Business, Innovation and Employment. The Welcome-to-School Study was supported by funding from Cure Kids (#4001), A+ Charitable Trust (6995), and Joyce Fisher Charitable Trust.

## Conflict of Interest

The authors declare that the research was conducted in the absence of any commercial or financial relationships that could be construed as a potential conflict of interest.

## Publisher's Note

All claims expressed in this article are solely those of the authors and do not necessarily represent those of their affiliated organizations, or those of the publisher, the editors and the reviewers. Any product that may be evaluated in this article, or claim that may be made by its manufacturer, is not guaranteed or endorsed by the publisher.
