# Supplementary material for: Caregiver Perception of Weight Status in 5-Year-Old Children From a Community of High Socioeconomic Deprivation in New Zealand
Source: Front Public Health. 2022 Jun 30;10:641418. doi: 10.3389/fpubh.2022.641418 (PMC9280196; doi:10.3389/fpubh.2022.641418)

Supplementary File 1

Questions included in the current study

When thinking about his/her weight, do you think ………… is?

- Underweight
- Normal weight
- Overweight
- Don't know

In general, how would you say …………… 's current health is?

- Excellent
- Very good
- Good
- Fair
- Poor
- Don't know

In your opinion, which of the following pictures of children looks about the same size as your child?

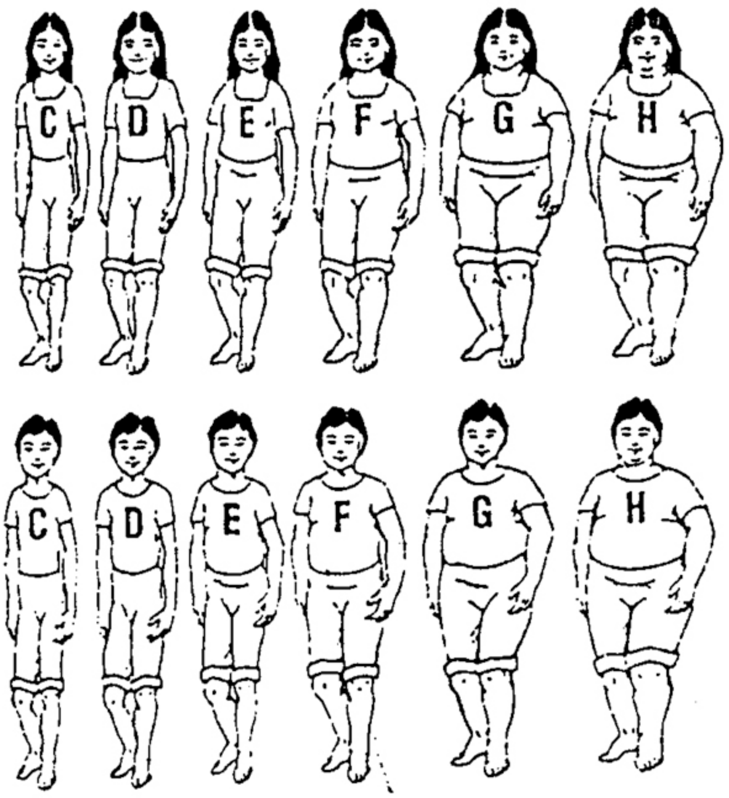

Figure reproduced with permission from Kersey et al. Overweight in Latino preschoolers: Do parental health beliefs matter? Am J Health Behav. 2010; 34: 340-8.

Supplementary Table 1

Caregiver perceived versus actual weight status of the child according to according to child's sex (girls n=49; boys n=57).

Child's sex	Caregiver perception	Child's weight status		
		Normal/underweight	Overweight	Obesity
**Girls**	**n**	25	11	13
	**Underweight**	3 (12%)	2 (18%)	nil
	**Normal**	22 (88%)	9 (82%)	10 (77%)
	**Overweight**	nil	nil	3 (23%)
**Boys**	**n**	21	12	24
	**Underweight**	3 (14%)	2 (17%)	2 (8%)
	**Normal**	18 (86%)	10 (83%)	15 (63%)
	**Overweight**	nil	nil	7 (29%)

Data are n (%). Child's body mass index (BMI) *z*-scores were standardised for age and sex as per World Health Organization standards (de Onis et al. Bull WHO 2007; 85: 660-7). Their BMI status was subsequently defined as: normal weight/underweight, BMI <85th percentile (*z*-score <1.036); overweight, BMI ≥85th to <95th percentile (*z*-score ≥1.036 and <1.645); and obesity, BMI ≥95th percentile (*z*-score ≥1.645) [Barlow. Pediatrics 2007; 120 (Suppl 4): S164-92].

Supplementary Figure 1

Caregiver's choice of silhouette and their child's body mass index (BMI) *z*-score according to ethnicity: Māori (n=19), Pacific (n=60), and Other (n=24).

Children's BMI *z*-scores were standardised for age and sex as per World Health Organization standards (de Onis et al. Bull WHO 2007; 85: 660-7). The two red lines parallel to the *x*-axis represent the thresholds for overweight and obesity, defined as: overweight, BMI ≥85th to <95th percentile (*z*-score ≥1.036 and <1.645); and obesity, BMI ≥95th percentile (*z*-score ≥1.645) [Barlow. Pediatrics 2007; 120 (Suppl 4): S164-92]. The associations between the child's BMI *z*-score and the caregiver's choice in the picture scale were examined using non-parametric Spearman's rank correlations, with results for a given ethnicity reported as the Spearman's coefficient (ρ) and the respective 95% confidence interval and p-value.

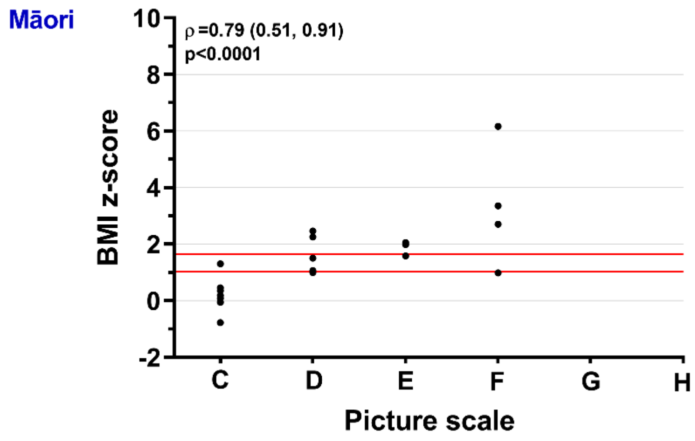

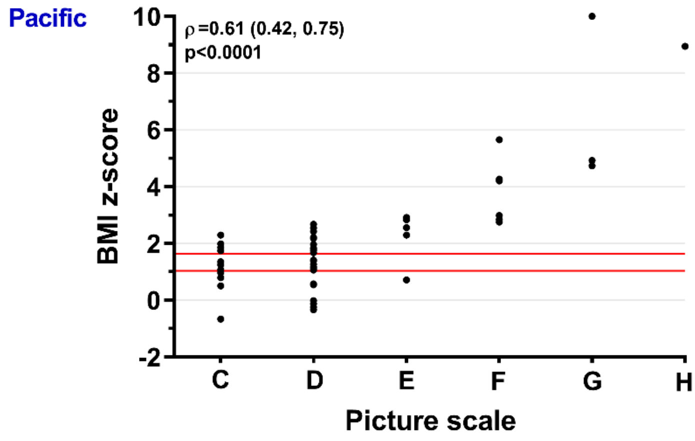

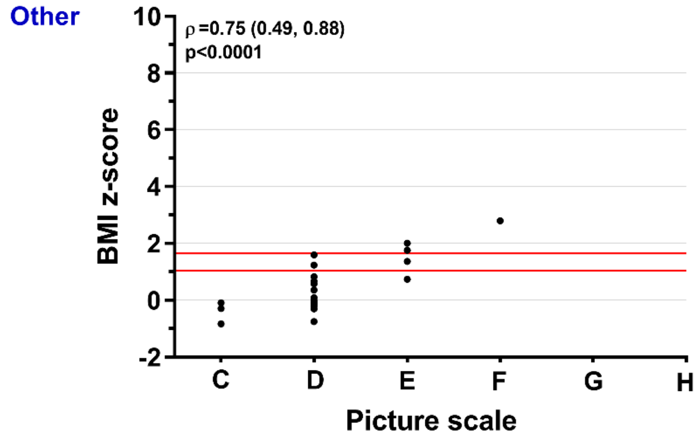

Supplementary Figure 2

Caregivers' choice of silhouette and their child's body mass index (BMI) *z*-score according to child's sex: females (n=49) and males (n=54).

Children's BMI *z*-scores were standardised for age and sex as per World Health Organization standards (de Onis et al. Bull WHO 2007; 85: 660-7). The two red lines parallel to the *x*-axis represent the thresholds for overweight and obesity, defined as: overweight, BMI ≥85th to <95th percentile (*z*-score ≥1.036 and <1.645); and obesity, BMI ≥95th percentile (*z*-score ≥1.645) [Barlow. Pediatrics 2007; 120 (Suppl 4): S164-92]. The associations between the child's BMI *z*-score and the caregiver's choice in the picture scale were examined using non-parametric Spearman's rank correlations, with results for each gender reported as the Spearman's coefficient (ρ) and the respective 95% confidence interval and p-value.

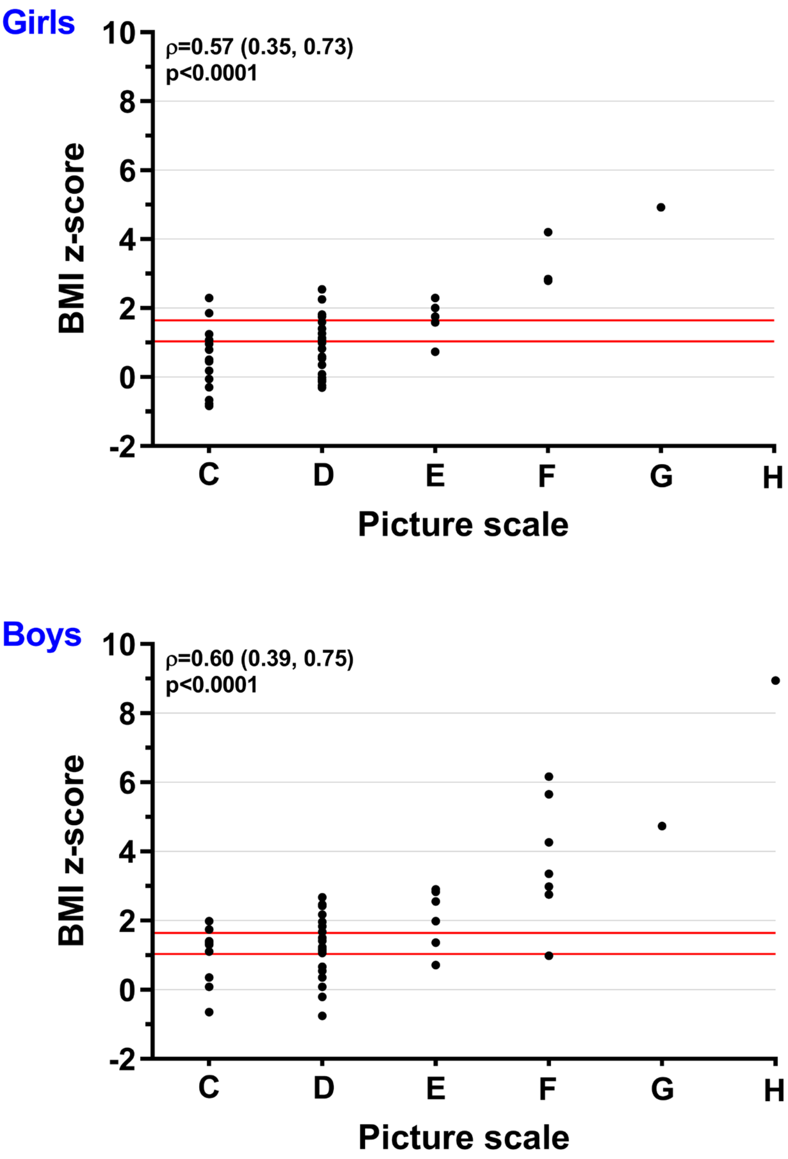

Supplementary Figure 3

Caregiver perception of child's health and child's BMI *z*-score (n=106).

Children's BMI z-scores were standardised for age and sex as per World Health Organization standards (25). The two red lines parallel to the x-axis represent the thresholds for overweight and obesity, defined as: overweight, BMI ≥85th to <95th percentile (z-score ≥1.036 and <1.645); and obesity, BMI ≥95th percentile (z-score ≥1.645) (26). Horizontal bars represent the median and the interquartile range.

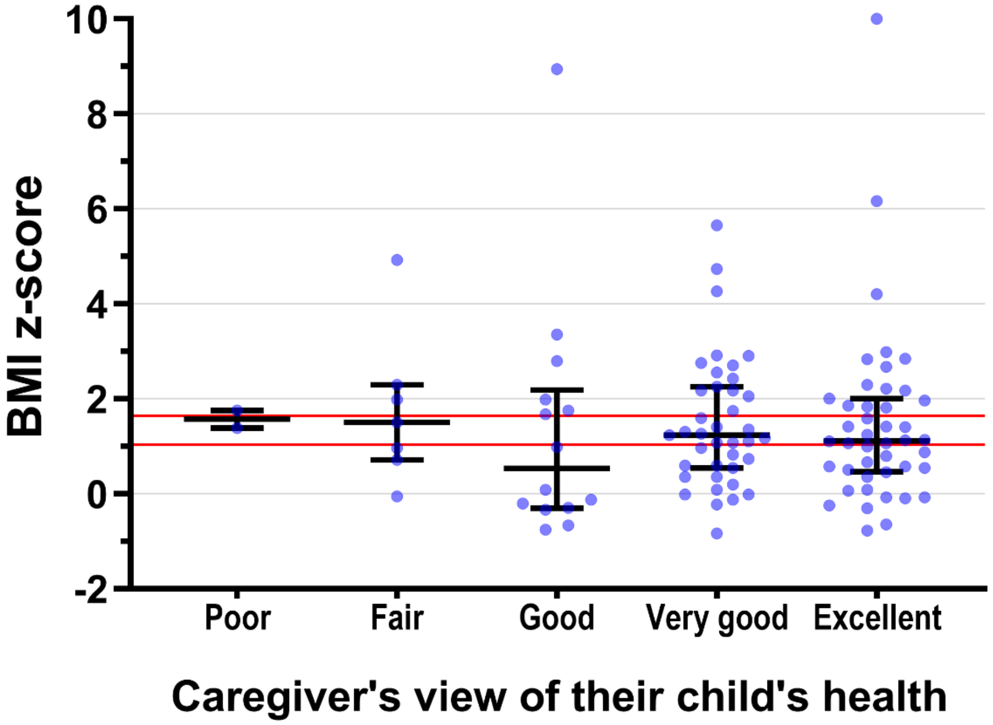

Supplement: Supplementary file 1 [file Data_Sheet_1.pdf]
